# Intravitreal Dexamethasone Implant for Patients With Central Retinal Vein Occlusion: Four-Year Outcomes in a Real-World Study

**DOI:** 10.7759/cureus.80391

**Published:** 2025-03-11

**Authors:** Anastasia Gkiala, Chrysa Agapitou, Eleni Dimitriou, Anna-Bettina Haidich, Tatiana Tziola, Panagiotis Theodossiadis, Asimina Mataftsi, Irini Chatziralli

**Affiliations:** 1 Department of Ophthalmology, Birmingham Midland Eye Center, Birmingham, GBR; 2 2nd Department of Ophthalmology, National and Kapodistrian University of Athens, Athens, GRC; 3 Department of Hygiene, Social-Preventive Medicine and Medical Statistics, School of Medicine, Faculty of Health Sciences, Aristotle University of Thessaloniki, Thessaloniki, GRC; 4 2nd Department of Ophthalmology, School of Medicine, Faculty of Health Sciences, Aristotle University of Thessaloniki, Thessaloniki, GRC; 5 2nd Department of Ophthalmology, National and Kapodistrian University of Athens School of Medicine, Athens, GRC

**Keywords:** dexamethasone, long-term outcomes, optical coherence tomography, retinal vein occlusion, visual acuity

## Abstract

Purpose: This study aims to present the long-term functional and anatomical outcomes of intravitreal dexamethasone (DEX) implant in patients with central retinal vein occlusion (CRVO) in real-world daily practice.

Methods: Retrospective study of consecutive patients with macular edema due to CRVO, treated with 0.7 mg DEX implant and had 48-month follow-up. Data on best-corrected visual acuity (BCVA) and central subfield thickness (CST) at months 12, 24, 36, and 48 after initiation of DEX implant treatment were collected from patients’ charts. Patient demographics and co-morbidities were also recorded, while potential factors affecting the final anatomical and functional outcomes were assessed.

Results: Thirty-one patients (31 eyes) received a mean number of 4.1±1.1 DEX implants and demonstrated significantly improved BCVA at all time-points of follow-up (p<0.001 for all comparisons). Accordingly, CST decreased significantly at all time-points of follow-up (p<0.001 for all comparisons). Treatment naïve patients were found to have lower BCVA at month 48 compared to those who had previously received intravitreal aflibercept, although there was no difference regarding CST between the two groups at month 48. When assessing factors that may predict the outcome, only naïve administration of treatment was found to have a negative correlation with BCVA at 48 months.

Conclusions: In this series of patients with macular edema secondary to CRVO, a demonstrable improvement in BCVA was recorded along with CST decrease at a long-term follow-up of four years. Only naïve treatment with DEX implant negatively correlated with visual acuity outcomes, but it was not confirmed at the multivariate analysis. Therefore, there was no evidence to support a predictive relationship between demographic and baseline anatomical factors and final BCVA and CST.

## Introduction

Retinal vein occlusion (RVO) is the second most common retinal vascular disease, after diabetic retinopathy, with a worldwide prevalence of 0.08% for central RVO (CRVO) and 0.4% for branch RVO (BRVO) without gender predisposition [1]. Atherosclerosis, inflammation, compression and vasospasm have been implicated in the pathogenesis of RVO, while risk factors include hypertension, diabetes mellitus, dyslipidemia, thrombophilia, systemic inflammatory disorders, smoking, ocular hypertension and an existing RVO in the contralateral eye [2-4].

Treatment of RVO does not focus on obstruction resolution, but on addressing its complications, mainly macular edema and retinal neovascularization due to ischemia. Since vascular endothelial growth factor (VEGF) has been involved in the pathogenesis of RVO [5], intravitreal anti-VEGF agents are the standard of care for RVO, halting vascular permeability and leakage, thus reducing macular edema [6-9]. Steroids are also used in the management of RVO, as inflammation is another key pathophysiological factor. Intravitreal triamcinolone acetonide injection [10] and intravitreal dexamethasone (DEX) implant have provided improvement in anatomical and functional outcomes in RVO-related macular edema [11]. Of note, the duration of macular edema prior to initiation of therapy is reversely associated with the response to therapy, while cataract and increased intraocular pressure (IOP) have been reported to be the main adverse effects of treatment [11]. Regarding the peak time of DEX implant efficacy, the SHASTA study found that patients with CRVO treated with DEX implant showed functional improvement one month after treatment, although severely limited after the fourth month, when re-injections were deemed necessary [12]. However, the approved dose regimen includes administration of DEX implant in an “as needed basis” with treatment intervals of less than or equal to six months, although earlier use has been tried in several studies [13].

In light of the above, this study aimed to evaluate the functional and anatomical results of DEX implant in patients with macular edema secondary to CRVO, providing long-term outcomes of 48 months. Moreover, potential predictive factors affecting the final visual and anatomical outcomes were assessed.

## Materials and methods

This retrospective study was a chart review of consecutive patients with macular edema secondary to CRVO diagnosed and treated at 2nd Department of Ophthalmology, University of Athens, Greece between January 1, 2016 and December 31, 2021. Macular edema was defined as central subfield thickness (CST) ≥320 μm. Inclusion criteria were treatment with 0.7 mg DEX implant (Ozurdex, Allergan), either as first-line treatment (naïve patients) or after switching from previous intravitreal aflibercept treatment (previously treated patients) and follow-up of at least 48 months. Patients with age-related macular degeneration, diabetic retinopathy, neovascularization at baseline, other retinal diseases except for CRVO, uncontrolled glaucoma (IOP>30 mmHg), history of vitrectomy, previous laser photocoagulation, uveitis, dense cataract and those who were lost to follow-up were excluded. The study adhered to the Declaration of Helsinki and written informed consent was obtained from all participants to use their data. No institutional review board approval was needed, since it was a retrospective study.

At baseline, all patients had undergone a thorough ophthalmic examination, including best-corrected visual acuity (BCVA) measurement using Snellen charts, slit-lamp examination, IOP measurement, dilated fundoscopy and spectral-domain optical coherence tomography (SD-OCT). Fundus fluorescein angiography (FFA) was performed at the physician’s discretion. SD-OCT and FFA were done using Spectralis HRA-OCT (Heidelberg Engineering, Heidelberg, Germany). BCVA was converted to logMAR (National Vision Research Institute, Victoria, Australia) for statistical analysis. We recorded demographic data and comorbidities in all patients.

All participants received 0.7 mg DEX implant at baseline and pro re nata (PRN) regimen, thereafter, leaving at least six months between injections. Retreatment criteria were the following: (i) decrease in BCVA ≥1 Snellen line, (ii) increase in CST≥50 μm and/or presence of intraretinal/subretinal fluid (IRF/SRF) on SD-OCT. If neovascularization was detected, panretinal photocoagulation was performed, as appropriate.

Patients were followed up every month in year 1 and at least every three months thereafter. At each visit, all patients underwent BCVA measurement and SD-OCT, while FFA was performed every six months for the first two years and then at the discretion of the physician. On SD-OCT, we measured the CST, and we assessed several biomarkers at baseline, such as the presence of IRF and SRF, the status of ellipsoid zone (EZ), the presence of hyperreflective foci (HF) and cysts. Ischemic type of CRVO was defined as retinal non-perfusion of greater than 10-disc diameter areas in FA, which could involve the periphery and/or the macula. Macular ischemia was defined as (i) foveal avascular zone (FAZ) more than 1,000 μm and (ii) broken perifoveal capillary rings at the borders of the FAZ with distinct areas of capillary non-perfusion within one disk diameter of the foveal center in the transit phase of FA.

We recorded the measurements of BCVA, OCT parameters (namely CST, IRF, SRF, HF, the presence of cysts, intact or disrupted EZ) and ischemia at baseline, as well as BCVA and CST at months 12, 24, 36, and 48 after initial DEX implant injection. The total number of DEX implant injections was also recorded.

The primary endpoints of our study include the mean change in BCVA and CST from baseline to month 48, while we also evaluated if difference in the main outcomes exists between naïve and previously treated patients. Predictive factors affecting visual acuity and CST difference at month 48 compared to baseline have also been assessed.

Statistical analysis

For the description of patients’ characteristics at baseline, mean ± standard deviation (SD) was used for continuous variables and counts with percentages for categorical variables. For the longitudinal comparisons of BCVA and CST between baseline and each time point, either the Wilcoxon matched pairs signed-ranks test or the paired t-test was used, depending on the normality of the variable distribution. Given that four comparisons were made (each follow-up time-point versus baseline), the level of statistical significance was set at 0.05/4=0.0125, according to the Bonferroni correction. Sub-analysis based on the administration of DEX implant in naïve or previously treated patients was also conducted. Comparisons between the two groups were performed using the Mann-Whitney U-Wilcoxon test or the Student’s t-test, as appropriate. For the assessment of factors that might predict final functional and anatomical outcomes, simple linear regression and subsequently multivariate linear regression analysis have been performed. The dependent factor was either the difference of BCVA between month 48 and baseline or the difference of CST between month 48 and baseline, while independent factors were the naïve administration of the DEX implant, the number of subsequent injections, as well as anatomical information, such as the presence of IRF, SRF, HF, cysts, the integrity of EZ, and the presence of macular ischemia on FFA. R studio (Posit PBC, Boston, MA) and SPSS 26.0 (IBM Corp., Armonk, NY) have been used for statistical analysis of the results.

## Results

The demographic and clinical characteristics of the study sample at baseline are shown in Tables 1, 2.

**Table 1:** Demographic and clinical characteristics at baseline for categorical variables.

Variable	n (%)
Gender	
Male	19 (61.3)
Female	12 (38.7)
Smoking	19 (61.3)
Hypertension	25 (80.6)
Diabetes mellitus	5 (16.1)
Dyslipidaemia	13 (41.9)
Cerebrovascular incident	7 (22.6)
Coagulation problems	4 (12.9)
Intraretinal fluid	31 (100)
Subretinal fluid	18 (58.1)
Hyperreflective foci	13 (41.9)
Cysts	24 (77.4)
Ellipsoid zone condition	
Intact	19 (61.3)
Disrupted	12 (38.7)
Macular ischemia	12 (38.7)

**Table 2:** Demographic and clinical characteristics at baseline for continuous variables. SD: standard deviation

Variable	Mean±SD
Age (years)	69.6±9.7
Best-corrected visual acuity (logMAR)	0.84±0.38
Central subfield thickness (μm)	497.9±88.7

The mean age of patients was 69.6±9.7 years, and 61.3% of patients were male. Regarding comorbidities, 25 out of 31 patients (80.6%) had hypertension, five patients (16.1%) diabetes mellitus, 13 patients (41.9%) dyslipidemia, and four patients (12.9%) coagulation problems, while seven out of 31 patients (22.6%) exhibited previous cerebrovascular incident.

In the study sample, 17 patients received DEX implant as first-line treatment, defined as “naïve” patients, while 14 patients were previously treated with intravitreal 2.0mg/mL aflibercept and were switched to DEX implant due to poor treatment response to aflibercept. The mean number of previous intravitreal anti-VEGF injections was 5.7±1.0. The mean number of intravitreal DEX implants in the 4-year follow-up was 4.1±1.1.

**Figure 1:**
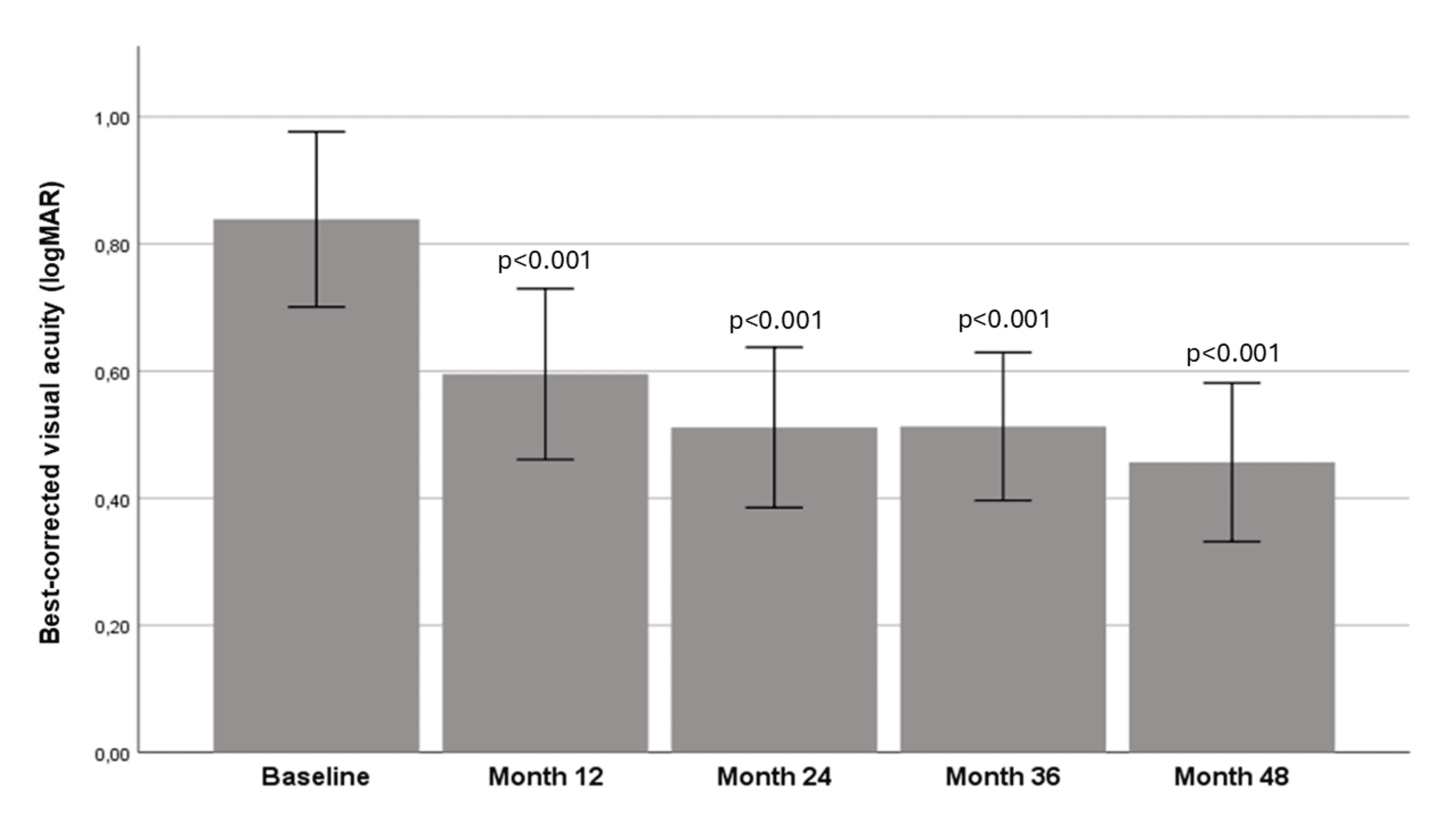
Evolution of best-corrected visual acuity over time. Mann-Whitney U-Wilcoxon test was used for all comparisons. P-value is depicted on the table, derived from the comparison of each time-point with baseline.

At baseline, the mean BCVA was 0.84±0.38 logMAR. There was a notable improvement in BCVA at month 12 (0.60±0.37 logMAR), month 24 (0.51±0.35 logMAR), month 36 (0.51±0.32 logMAR) and month 48 (0.46±0.35 logMAR) compared to baseline (p<0.001 for all comparisons). Figure 1 shows the evolution of BCVA over time.

**Figure 2:**
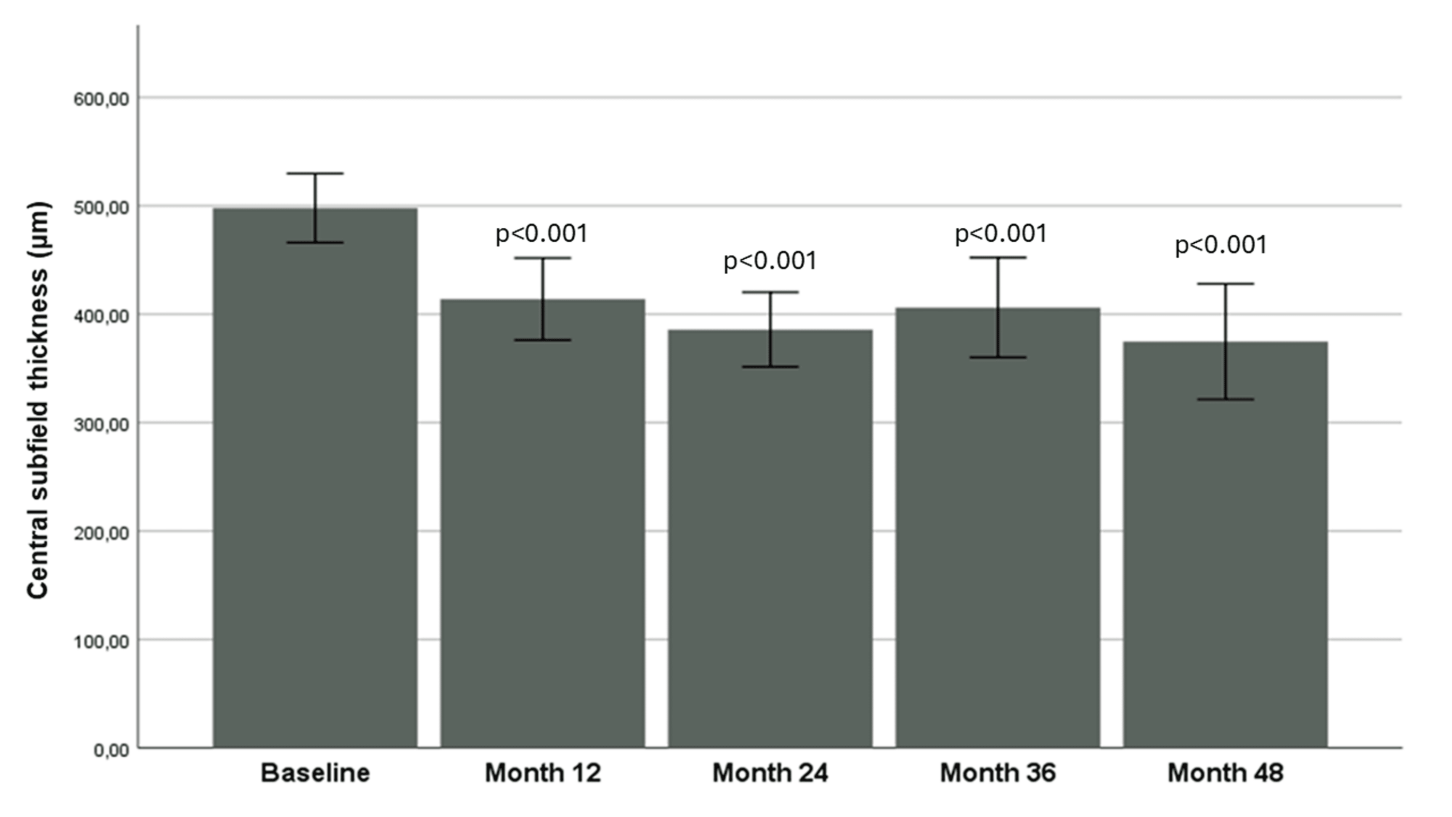
Evolution of central subfield thickness over time. Mann-Whitney U-Wilcoxon test was used for all comparisons. P-value is depicted on the table, derived from the comparison of each time-point with baseline.

Accordingly, at baseline, the mean CST was 497.9±88.7 μm. It decreased remarkably at month 12 (414.0±105.4 μm), month 24 (385.9±95.7 μm), month 36 (406.2±128.2 μm), and month 48 (374.7±148.4 μm) compared to baseline (p<0.001 for all comparisons). Figure 2 shows the progression of CST over time. A sub-analysis between DEX “naïve” patients and patients “previously treated with aflibercept” showed that the change in BCVA from baseline to month 48 did not differ in the two groups (p=0.826), as well as the change in CST from baseline to month 48 (p=0.383).

**Figure 3:**
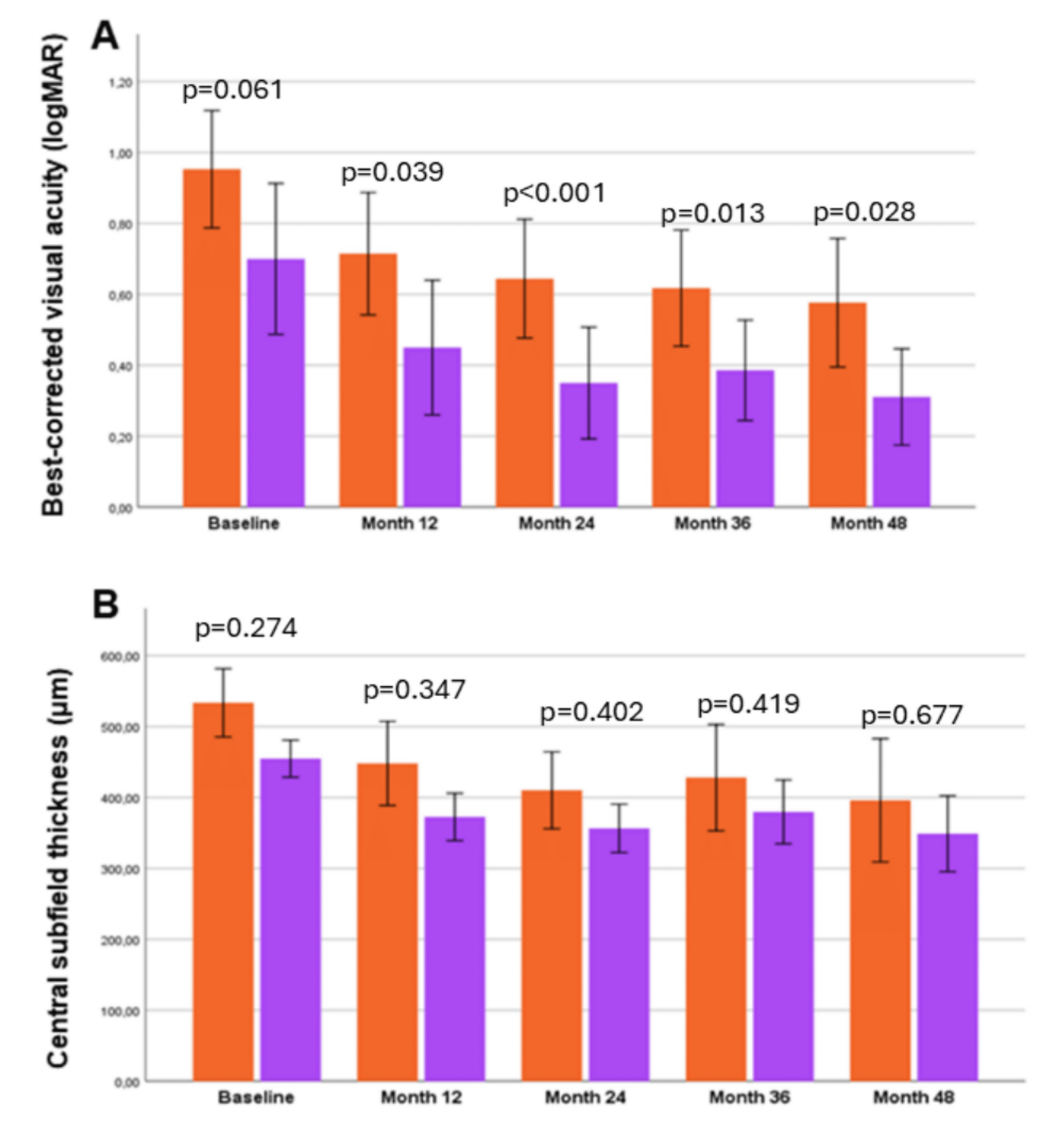
(A) Mean best-corrected visual acuity (logMAR) between naïve (orange bars, n=17) and previously treated (purple bars, n=14) patients at the four follow-up time-points; (B) Mean central subfield thickness between naïve (orange bars) and previously treated (purple bars) patients at the four follow-up time-points. Mann-Whitney U-Wilcoxon test was used. P-value is depicted on the table, derived from the comparison between the two groups.

Figures 3A, 3B show the comparison between naïve and previously treated eyes in BCVA and CST over time.

Regarding predictive factors for visual and anatomical outcomes, only “naïve” administration of DEX implant was negatively correlated to BCVA at month 48 (p=0.031) at the univariate analysis (Table 3). However, results of the multivariate linear regression analysis showed that none of the examined factors was associated with BCVA and CST at month 48.

**Table 3:** Correlation between demographic and anatomical baseline features and final visual acuity and final central subfield thickness. Linear regression analysis was used. * denotes statistical significance.

Variables	Best-corrected Visual Acuity at month 48	Central Subfield Thickness at month 48
Age	p=0.547	p=0.078
Sex	p=0.199	p=0.716
Smoking	p=0.148	p=0.147
Hypertension	p=0.665	p=0.593
Diabetes mellitus	p=0.422	p=0.835
Dyslipidaemia	p=0.851	p=0.483
Cerebrovascular indicent	p=0.298	p=0.198
Coagulation problems	p=0.734	p=0.629
Naïve patients	p=0.031*	p=0.388
Subretinal fluid	p=0.708	p=0.401
Hyperreflective foci	p=0.811	p=0.579
Cysts	p=0.995	p=0.052
Ellipsoid zone condition	p=0.421	p=0.278
Macular ischemia	p=0.392	p=0.347

There were no safety issues with the use of intravitreal DEX implant in this study. Only three out of 31 patients (9.7%) exhibited high IOP during the follow-up period and were well-controlled with anti-glaucoma drops. No filtering surgery was needed.

## Discussion

 The principal message of this study is that intravitreal DEX implant was shown to be effective in patients with macular edema secondary to CRVO, leading to significant improvement in BCVA and decrease in CST at the long-term follow-up of 48 months, with a mean number of 4.1±1.1 implants. Of note, the mean change in BCVA and CST at month 48 compared to baseline did not differ between patients who received DEX implant as first-line treatment and those who were previously treated with intravitreal aflibercept injections before switching to DEX implant.

 Intravitreal DEX implant acts mainly through the anti-inflammatory properties of cortisone. It is believed to reduce vascular permeability, halt recruitment of inflammatory cells, as well as the deposition of fibrin, while stabilizing intra-cell junctions and suppressing VEGF, prostaglandin and other cytokine synthesis [14,15]. This implant is made of a biodegradable material (lactic acid copolymer and glycolic acid) and 700μg micronized DEX, introduced into the vitreous cavity and releases its active substance gradually through a pars plana puncture created at insertion [14].

The efficacy of DEX implant in the management of RVO-related macular edema is underlined by a number of real-world studies, providing evidence that it increases final BCVA and decreases retinal thickness [16-21]. More specifically, when compared to anti-VEGF agents, and although not considered first-line treatment for RVO-related macular edema, steroid implants were found to result in better BCVA at 1-month follow-up, although no overall difference in mean BCVA between the two groups was observed [22]. Similar results were found by Guignier et al. [23], although these authors report superiority of anti-VEGF agents not only regarding BCVA but also concerning CST. Moreover, steroid implants were found to have a higher possibility of adverse effects, such as elevated IOP and cataract formation, when compared to anti-VEGF factors, although no difference exists regarding serious adverse effects, such as serious cardiac and vascular disorders, eye or psychiatric disorders [24]. Furthermore, the efficacy of DEX implants in refractory anti-VEGF cases has been shown in other studies [25]. In our study, a slight decrease in BCVA and an increase in CST were found at month 36. However, BCVA increased at month 48 with a concurrent remarkable decrease in CST. A lower number of DEX implants were administered in our patients between month 24 and 36, due to unavailability of implants in the European market for several months, and this may have contributed to the above findings. The unavailability of the DEX implant during this period may reflect real-world challenges faced by clinicians and patients, affecting also treatment consistency and outcomes, since supply issues may lead to undertreatment, which is a crucial point regarding the efficacy of treatment. In such cases, alternative treatment modalities should be used, such as intravitreal anti-VEGF agents or intravitreal fluocinolone acetonide implant. It is noteworthy that the follow-up period of our study is one of the longest reported in the existing literature [16-21].

Other studies have demonstrated that combined DEX implant and anti-VEGF treatment therapy had better results than monotherapy, not only regarding visual acuity, but also retinal thickness of patients with RVO-related macular edema. The beneficial effect also lasted longer than in patients who received single treatment, but were associated with higher risk of adverse effects, such as increased IOP [26,27]. In our study, there were no safety issues and although about 10% of patients exhibited increased IOP, this was controlled with anti-glaucoma drops and no filtering surgery was needed.

Regarding predictive factors for the final visual outcome after treatment of RVO-related macular edema with intravitreal DEX implants, the presence of foveal serous retinal detachment and macular ischemia are negatively correlated with visual outcomes, while high baseline BCVA, the presence of non-chronic macular edema and the integrity of EZ have been proven to have a positive association [21,28]. These factors were investigated in our study but yielded no association with BCVA or CST.

Potential limitations of our study pertain to the small sample size and to its retrospective design, which may limit the generalizability of our findings. Additionally, the lack of control group and the inconsistency in follow-up intervals may introduce biases. However, this is a real-life study with a particularly long-term follow-up, of 48 months.

## Conclusions

In conclusion, our study, despite its limited sample size, was able to demonstrate improvement in BCVA and reduction in CST between baseline and the four time-points of the follow-up period at month 12, 24, 36, and 48, with a mean number of 4.1±1.1 implants. Treatment naïve patients had lower final BCVA compared to patients previously treated with anti-VEGF agents. No association was found between various OCT biomarkers at baseline and final BCVA or CST at month 48.
